# Functional Relevance of AcrB Trimerization in Pump Assembly and Substrate Binding

**DOI:** 10.1371/journal.pone.0089143

**Published:** 2014-02-14

**Authors:** Wei Lu, Meng Zhong, Qian Chai, Zhaoshuai Wang, Linliang Yu, Yinan Wei

**Affiliations:** Department of Chemistry, University of Kentucky, Lexington, Kentucky, United States of America; Centre National de la Recherche Scientifique, Aix-Marseille Université, France

## Abstract

AcrB is a multidrug transporter in the inner membrane of *Escherichia coli*. It is an obligate homotrimer and forms a tripartite efflux complex with AcrA and TolC. AcrB is the engine of the efflux machinery and determines substrate specificity. Active efflux depends on several functional features including proton translocation across the inner membrane through a proton relay pathway in the transmembrane domain of AcrB; substrate binding and migration through the substrate translocation pathway; the interaction of AcrB with AcrA and TolC; and the formation of AcrB homotrimer. Here we investigated two aspects of the inter-correlation between these functional features, the dependence of AcrA-AcrB interaction on AcrB trimerization, and the reliance of substrate binding and penetration on protein-protein interaction. Interaction between AcrA and AcrB was investigated through chemical crosslinking, and a previously established *in vivo* fluorescent labeling method was used to probe substrate binding. Our data suggested that dissociation of the AcrB trimer drastically decreased its interaction with AcrA. In addition, while substrate binding with AcrB seemed to be irrelevant to the presence or absence of AcrA and TolC, the capability of trimerization and conduction of proton influx did affect substrate binding at selected sites along the substrate translocation pathway in AcrB.

## Introduction

AcrB is a multidrug transporter in the inner membrane of *Escherichia coli* [1]–[3]. It is a secondary transporter, harvesting the proton gradient across the inner membrane to drive the efflux of an array of structurally different compounds out of the cell [1]–[3]. AcrB exists and functions as a homotrimer and forms a tripartite complex with outer membrane protein TolC and membrane fusion protein AcrA. Together they form an efflux machinery that spans both layers of membranes and the periplasmic space. This AcrA-AcrB-TolC complex and its homologues are major players in multidrug resistance in Gram-negative bacteria [4]. AcrB is the engine of the complex and determines substrate specificity. Crystal structures of AcrB have been obtained both in the substrate free and bound states [5]–[17]. The pathway of substrate entry and exit has been proposed based on these structures and subsequent mutational studies [17]–[25]. Recently, Nikaido and co-workers have mapped the substrate translocation pathway in AcrB through a combination of site-directed mutagenesis and fluorescent labeling [26], [27].

Functional features that are critical to AcrB drug efflux include the proton translocation via the proton relay pathway, substrate binding and migration through the substrate translocation pathway, and AcrB trimerization and the interaction with AcrA and TolC to form a sealed exit path across the periplasm and outer membrane. Substrate extrusion requires all features to operate properly. In this study we investigated the effects of each individual aspect, namely proton relay, interaction with AcrA/TolC, and trimerization, on substrate binding. While disruptions of interaction with AcrA/TolC and proton relay are easy to realize experimentally, it was more complicated to create monomeric AcrB. In a recent study we have constructed such a mutant, AcrB_Δloop_, which provided us a tool to investigate the functional role of AcrB trimerization on substrate binding and interaction with its functional partner AcrA [28].

**Figure 1:**
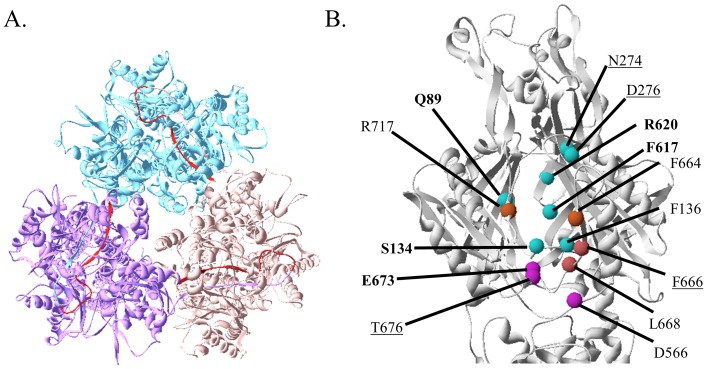
Structure of AcrB. **A.** Top view of AcrB trimer from the periplasmic side of the membrane. Subunits are color-coded with residues 211 to 227 colored in red. **B.** Periplasmic domain of AcrB with sites tested in this study highlighted by spheres at the positions of their Cα position. Cα spheres were colored following the scheme established in an earlier publication with brown, purple, and teal representing entrance to the external cleft, bottom of the external cleft, and the deep drug binding pocket, respectively [26]. Structure was created from 2DHH.pdb [8].

To create AcrB_Δloop_, residues 211 to 227 in AcrB, which are part of a long extended loop that is critical for inter-subunit interaction, were deleted (Figure 1A). Residue 210 was directly connected to residue 228. The rationale behind the design was that since this loop is not involved in the packing of the tertiary structure of AcrB, changes made on the loop should not have a significant impact on the folding of each subunit. As a summary of the previous study, we first confirmed AcrB_Δloop_ expressed to a level similar to wild type AcrB but was completely non-functional [28]. AcrB_Δloop_ could be purified similarly to the wild type AcrB with comparable yield. The secondary structure component of the mutant was comparable with that of the wild type protein as revealed by the circular dichroism (CD) spectra. Heat denaturation of the two proteins was monitored at 222 nm using CD and the two curves superimposed well onto each other, indicating similar secondary structure stability. Furthermore, we confirmed AcrB_Δloop_ existed as a monomer using Blue Native (BN)-PAGE, while wild type AcrB is a trimer. We have also confirmed that loop truncation did not have a significant effect on the overall tertiary conformation of the periplasmic domain using a disulfide trapping based method [28], [29].

In this study we examined the relative accessibility of the substrate translocation pathway under three conditions: in AcrB_Δloop_ which is monomeric; in AcrB_D407A_ which is defective in proton translocation; and in a *ΔacrABtolC* knockout strain where both AcrA and TolC are absent. Although no drug efflux could occur under all three conditions, the levels of “damage” to the efflux machinery were different. In the knockout strain the structure of AcrB trimer is intact and is capable of drug extrusion. In AcrB_D407A_ the protein remains as a trimer and still interacts with AcrA and TolC, although proton relay is disrupted [30]–[32]. While in AcrB_Δloop_, the protein dissociates into monomers. We compared substrate binding and penetration in AcrB under these three conditions. In addition, we examined the interaction of AcrB_Δloop_ with the functional partner AcrA.

## Results

### Substrate Binding in AcrB_Δloop_

We choose to use the *in vivo* fluorescent labeling method developed by Nikaido and co-workers due to its unique advantage that labeling is conducted in live cell cultures with AcrB still embedded in the cell membrane. Therefore, the result will better reflect the actual state of the protein and will not be affected by artifact from detergent solubilization and protein purification [26], [27]. It has been demonstrated that labeling is highly specific to Cys in the translocation pathway. Cys introduced at random locations on the surface of AcrB were not labeled. To investigate the binding and penetration of substrate in AcrB_Δloop_, we replaced the two intrinsic Cys with Ala to create _CL_AcrB_Δloop_ and chose to label 14 sites along the substrate translocation pathway based on two considerations: First, we expect the level of labeling in _CL_AcrB_Δloop_ to be equal or weaker than the level of labeling in trimeric _CL_AcrB. Therefore, we chose sites that are labeled strongly in _CL_AcrB so a clear difference could be observed if labeling were significantly reduced in _CL_AcrB_Δloop_. Second, we selected sites that are distributed evenly along the pathway. The locations of the chosen residues are shown in Figure 1B. These residues were color-coded, in which F664, F666, L668 and R717 were at the entrance of the external cleft, D566, E673 and T676 were part of the bottom of the cleft, and Q89, S134, F136, N274, D276, F617, and R620 were in a deep drug binding pocket. For each site, a single Cys mutation was introduced into either trimeric _CL_AcrB or monomeric _CL_AcrB_Δloop_ and their labeling was examined in BW25113*ΔacrB* strain.

**Figure 2:**
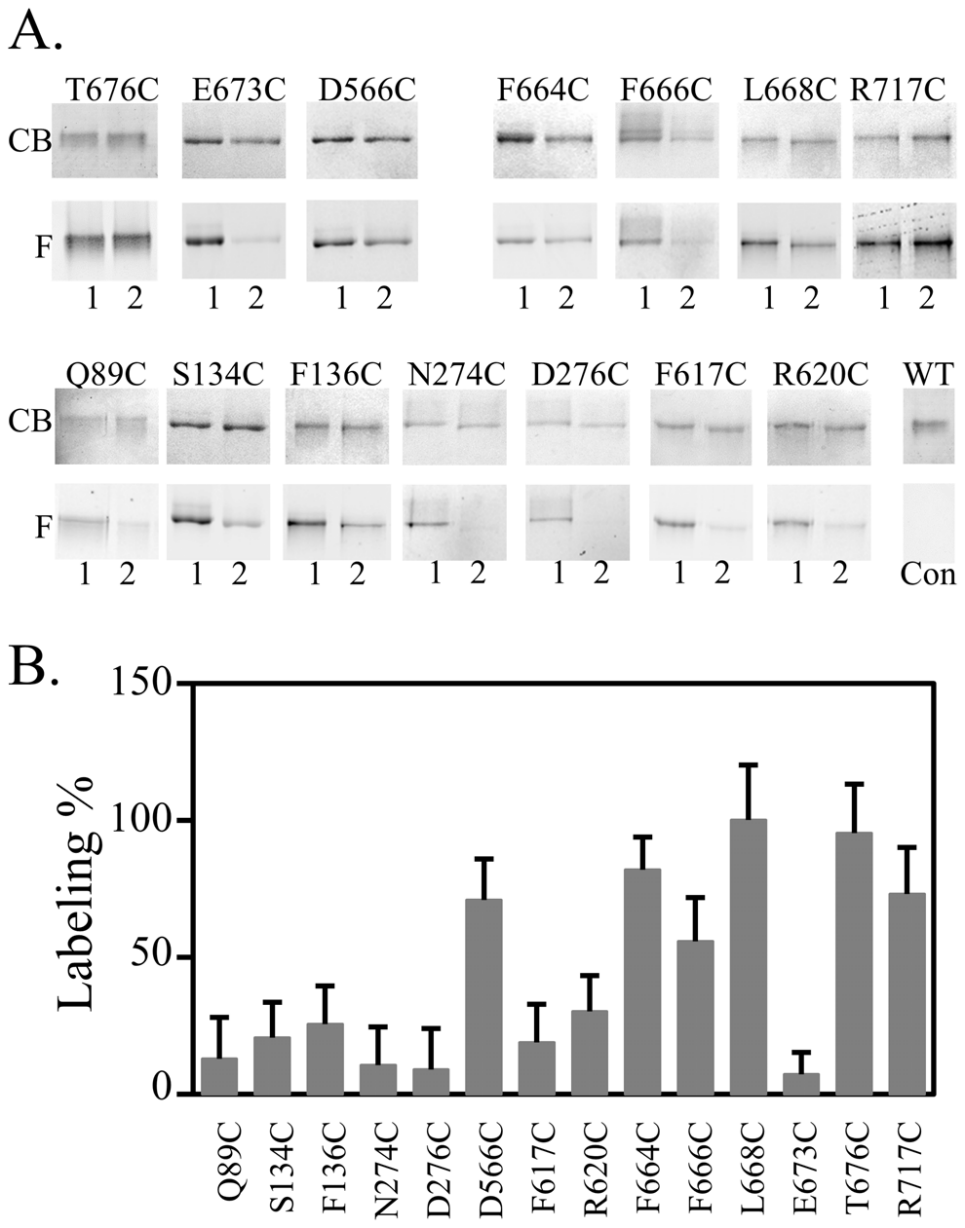
Comparison of substrate accessibility of residues lining up the substrate translocation pathway in monomeric and trimeric AcrB. **A.** Representative gel images. For each position tested, the bottom panel (F) is the fluorescence image before staining and the top panel (CB) is the image of the same gel after Coomassie blue stain. Lane 1 and 2 were mutants containing Cys at the indicated site on the background of _CL_AcrB and _CL_AcrB_Δloop_, respectively. _CL_AcrB without introduced Cys was used as the negative control (lane WT, con) to confirm the lack of non-specific labeling. **B**. Relative percentage of labeling for each site in _CL_AcrB_Δloop_ relative to _CL_AcrB. Each experiment was performed three times. The average value and standard deviation were shown.

Fluorescent labeling and purification was conducted as described in Materials and Methods. Briefly, BW25113*ΔacrB* cells containing plasmids encoding different AcrB mutants were incubated with Bodipy-FL-maleimide. After the removal of excess dye, cells were lysed for protein purification using metal affinity chromatography. Purified samples were analyzed using SDS-PAGE. Representative gel images taken under fluorescent light before Coomassie staining (F) and under white light after staining (CB) were shown in Figure 2A. The ratio of labeling at each site was obtained by dividing the concentration-normalized fluorescence signal of the _CL_AcrB_Δloop_ sample by the concentration-normalized fluorescence signal of the _CL_AcrB sample as described in Materials and Methods (Figure 2B). For several sites, including 566C, 664C, 666C, 668C, 676C, and 717C, the level of fluorescent labeling in _CL_AcrB_Δloop_ were comparable to the level of labeling in _CL_AcrB. For the rest of the sites tested, levels of labeling in _CL_AcrB_Δloop_ were significantly lower than those in _CL_AcrB.

### Substrate Binding in AcrB_D407A_

In 2006, two research groups independently reported the asymmetric structure of AcrB trimer, which supported a conformational cycling model for drug transport [8], [12]. In the asymmetric trimer, one subunit in each trimer bound a drug molecule. The conformation of each subunit was different and the binding site located in the periplasmic domain of AcrB. Based on those different conformations, the subunits were designated as loose (access), tight (binding) and open (extrusion) state, respectively. A conformational rotation mechanism for drug export has been proposed. A substrate binds with the subunit at the loose state, rotates through the tight, and then open state before being pumped out of the cell. The asymmetric subunits represent different stages of the pumping cycles. Sennhauser et al. used a Designed Ankirin Repeat Protein (DARPin) to co-crystallize with AcrB and obtained the asymmetric structure at the highest resolution of 2.5 Å [13]. The stoichiometry of AcrB-DARPin complex was 3∶2, and the DARPin was shown to stabilize the intermediates conformation in the transport cycles which supported a rotary mechanism for AcrB drug transport. Energy required to drive this conformational rotation derives from proton translocation down its concentration gradient across the inner membrane. Residue D407 in AcrB is a critical residue on the proton relay pathway. AcrB_D407A_ was completely inactive due to the disruption of proton translocation [30], [33]. In AcrB_D407A_ each protomer might be “frozen” into a fixed conformation rather than rotating through three conformations. Other than that the overall structure of AcrB_D407A_ was very similar to the structure of wild type AcrB [30]. It remains as a tightly associated trimer and interacts with AcrA and TolC [30]–[32]. In a previous study the relative levels of labeling in _CL_AcrB_D407A_ as compared to _CL_AcrB had been reported for four sites, N274, D276, F666, and T676, underlined on Figure 1B [26]. Among the sites tested, _CL_AcrB_N274C/D407A_, _CL_AcrB_D276C/D407A_ and _CL_AcrB_F666C/D407A_ were labeled at the levels of 61%, 42% and 47% of the corresponding controls _CL_AcrB_N274C_, _CL_AcrB_D276C_, and _CL_AcrB_F666C_, respectively. No difference in the labeling of _CL_AcrB_T676C_ and _CL_AcrB_T676C/D407A_ was observed.

**Figure 3:**
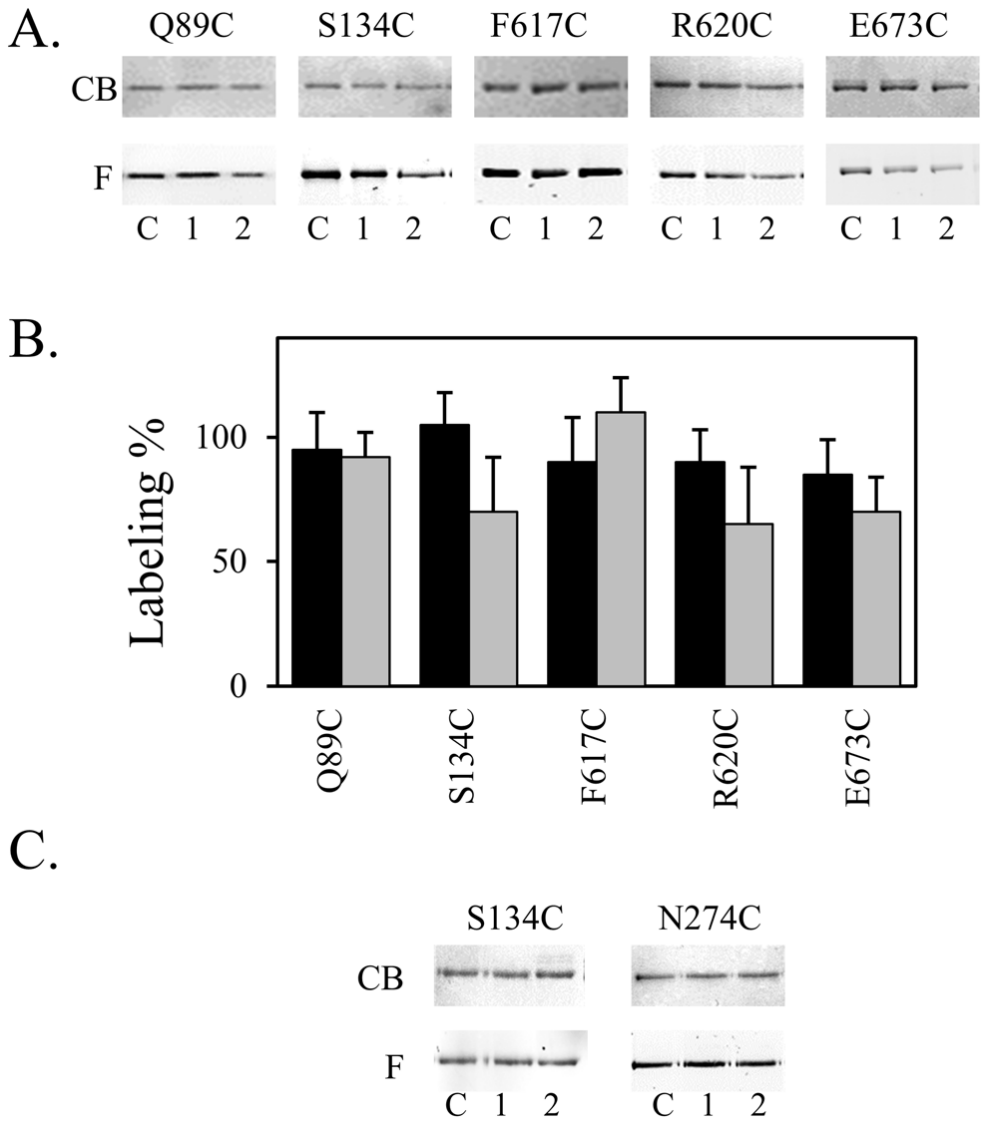
Substrate accessibility of residues lining up the substrate translocation pathway in the triple knockout strain and in AcrB_D407A_. **A.** Representative gel images. For each position tested, the bottom panel (F) is the fluorescence image before staining and the top panel (CB) is the image of the same gel after Coomassie blue stain. Labeling of mutants containing Cys at the indicated site on the background of _CL_AcrB in MG1655*ΔacrB* (lane C) or MG1655*ΔacrABtolC* (lane 1), and _CL_AcrB_D407A_ in MG1655*ΔacrB* (lane 2). **B.** Relative percentage of labeling for each site in *acrABtolC* knockout strain as compared to *acrB* knockout strain (black), and in _CL_AcrB_D407A_ as compared to in _CL_AcrB (grey). Each experiment was performed three times. The average value and standard deviation were shown. **C.** Representative gel images of labeling in the absence (lane C) or presence of over-expressed AcrA (lane 1) or TolC (lane 2). Gels were labeled similarly as above.

We found that the levels of labeling of F666 and T676 in _CL_AcrB_Δloop_ were comparable to the levels of labeling in _CL_AcrB_D407A_. However, the labeling of N274 and D276 was much less in the monomeric mutant. Furthermore, we tested the labeling of five additional sites in _CL_AcrB_D407A_, including Q89C, S134C, F617C, R620C, and E673C. These sites were chosen because they were labeled significantly less in _CL_AcrB_Δloop_ as compared to in _CL_AcrB. For sites that were labeled similarly in monomeric AcrB as compared to wild type AcrB, we expect the level of labeling would not be affected by the gentler change as a result of the D407A mutation. Labeling of _CL_AcrB and _CL_AcrB_D407A_ were conducted in MG1655*ΔacrB* strain. Representative gel images taken under fluorescent light before Coomassie staining (F) and under white light after staining (CB) were shown in Figure 3A (Lane 2). Labeling of the corresponding sites in _CL_AcrB was also conducted in parallel and loaded to the same gel (Figure 3A, lane C). The ratio of labeling at each site was obtained by dividing the concentration-normalized fluorescence signal of the _CL_AcrB_D407A_ sample by the concentration-normalized fluorescence signal of the _CL_AcrB sample as described in materials and method (grey bars, Figure 3B). Overall the levels of labeling in _CL_AcrB_D407A_ of all sites tested were significantly higher than the level of labeling in _CL_AcrB_Δloop_. When compared to the level of labeling in _CL_AcrB, little difference could be observed for sites Q89C and F617C, and 30–40% differences could be observed for S134C, R620C and E673C.

### Substrate Binding in the Absence of AcrA and TolC

AcrB function as a component of the protein complex together with AcrA and TolC. No substrate efflux could occur in the absence of either AcrA or TolC. To further investigate the correlation between drug efflux and substrate binding, we examined the labeling of selected sites in a triple knockout strain lacking chromosomally encoded AcrAB and TolC. In this case while not functional, the structure of AcrB is intact. We labeled the same panel of sites as in the case of _CL_AcrB_D407A_ in MG1655*ΔacrB* (Figure 3A, lane C) and MG1655*ΔacrABtolC* (Figure 3A, lane 1). Labeling of each site in all three samples, including the control _CL_AcrB, _CL_AcrB_D407A_, and _CL_AcrB in the triple knockout strain, were actually conducted in parallel for a better comparison. The ratio of labeling at each site was obtained similarly as described above (black bars, Figure 3B). Overall the levels of labeling in the triple knockout strain of all sites tested were very similar to the levels of labeling in the control sample, indicating that the absence of AcrA and TolC did not have a significant effect on substrate binding and penetration in AcrB.

One potential cause that could have resulted in the lack of difference in strains of MG1655 *ΔacrB* and MG1655*ΔacrABtolC* is the mismatch of expression levels–it is possible that since AcrB was expressed from a plasmid while AcrA and TolC were from the chromosomal DNA, the final expression levels of AcrB would be higher than the level of AcrA and TolC. Therefore, AcrA and TolC could be deficient even under the control condition. The chromosomal expression levels of AcrA, AcrB, and TolC in *E. coli* strain ZK4 have been examined and the relative molar ratios of these three proteins were estimated to be 5000–7000∶500∶1500 [34]. We have also determined the expression level of AcrB from plasmid pQE70-AcrB under the basal condition to be roughly 16 times of the chromosomal expression level [35]. When AcrB was expressed from a plasmid there is likely a shortage of AcrA and TolC. Therefore, we co-expressed AcrA or TolC with AcrB by transforming two compatible plasmids into MG1655*ΔacrB* and examined the effect on labeling at two sites, N274C and S134C. These two sites were chosen to represent sites for which labeling were affected by both D407A mutation and the trimer dissociation, and thus are expected to be more sensitive to changes of AcrA and TolC concentrations. As shown in Figure 3C, the over-expression of AcrA and TolC had no observable effect on the level of fluorescent labeling, further confirmed our previous observation that the presence or absence of AcrA and TolC did not have a significant effect on substrate access to the translocation pathway in AcrB.

### Interaction of AcrB_Δloop_ with Functional Partner AcrA

**Figure 4:**
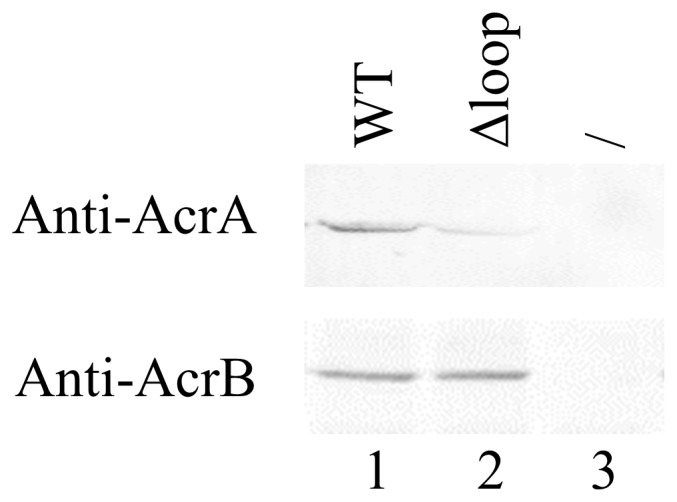
Cross-linking of AcrA with AcrB (1ane 1, WT) and AcrB_Δloop_ (lane 2, Δloop). In the control sample (lane 3,/) the empty vector was used in the transformation. The top and bottom panels are representative blots detected using the anti-AcrA and anti-AcrB antibodies, respectively.

Interaction of AcrA with wild type trimeric AcrB and monomeric AcrB_Δloop_ was examined using an established protocol in literature [36], [37]. *E. coli* cells containing AcrB or AcrB_Δloop_ were treated with a chemical cross-linker dithiobis succinimidyl propionate (DSP), which has been shown to form covalent linkages between wild type AcrA and AcrB in *E. coli* cells. After cross-linking, cells were lysed and proteins capable of binding to Ni-NTA resin were purified. AcrA, in the absence of AcrB, could not be purified from the cell lysate (Figure 4, lane 3). In the presence of AcrB, a significant AcrA band was visible, indicating that the interaction between AcrA and AcrB was critical for the detection of the AcrA band in the purified sample (Figure 4, lane 1). Finally, when AcrB was replaced with the monomeric mutant AcrB_Δloop_, the level of co-purified AcrA decreased drastically, indicating that the interaction between AcrB_Δloop_ and AcrA was weaker than the interaction between the wild type proteins (Figure 4, lane 2).

### Effect of Cys Mutation on AcrB Activity

To evaluate the effect of Cys mutation on the efflux activity of AcrB, we measured the activity of each mutant using a drug susceptibility assay. The minimum inhibitory concentrations (MICs) for two well established AcrB substrates, erythromycin and novobiocin, were listed in Table 1. BW25113*ΔacrB* strains containing a plasmid encoding the wild type AcrB (pQE70-AcrB) or the empty vector (pQE70) were used as the positive and negative controls, respectively. The mutants displayed a broad range of activities. This is not surprising as mutations of residues lining up the substrate translocation pathway may impact substrate efflux, some more than others.

**Table 1:** MIC of BW25113*ΔacrB* strains containing plasmids encoding the indicated AcrB constructs and a summary of labeling result.

AcrB construct		MIC (µg/ml)		Significant Change in Labeling?		
		erythromycin	novobiocin	Δloop	D407A	*ΔacrAtolC*
WT		80	160			
/*		5	5			
Entrance of the external cleft						
F664C	20		40	No		
F666C	40		10	No		
L668C	80		80	No		
R717C	5		10	No		
Bottom of the cleft						
D566C	10		10	No		
E673C	80		160	Yes	Yes	No
T676C	80		80	No		
Deep binding pocket						
Q89C	80		160	Yes	No	No
S134C	80		160	Yes	Yes	No
F136C	80		160	Yes		
N274C	80		80	Yes	Yes**	
D276C	80		160	Yes	Yes**	
F617C	40		40	Yes	No	No
R620C	80		80	Yes	Yes	No

*: Vector pQE70 was used to transform BW25113*ΔacrB* and used as the negative control.

**: According to reference 26.

## Discussion

The triplex efflux system AcrAB-TolC is a key player in multidrug resistance in *E. coli*. In this large protein complex, AcrB is the component that first takes up substrates from the periplasm and/or inner membrane of the cell [1]–[3]. Many residues in AcrB have been found to make direct contact with substrate and line up the drug translocation pathway [26], [27]. For effective efflux, a substrate molecule has to bind and go through the drug translocation pathway in the periplasmic domain of AcrB. But how much does substrate binding and penetration rely on active efflux? To answer this question, we studied fluorescent labeling of sites lining up the substrate translocation pathway under three conditions devoid of active efflux. These conditions differ in the level of structure impairment in AcrB, and the observed level of labeling in response of structure changes differed for different sites. Labeling of 14 sites was examined in this study, and their data are summarized in Table 1.

The most surprising discovery is the lack of significant response of the level of labeling to the absence of AcrA and TolC. In other words, substrate can bind and enter the translocation pathway of AcrB even without active drug efflux. As discussed above, the three subunits in an AcrB trimer adopt different conformations that are intrinsically not equally accessible by the substrates. The observation that all sites tested could be labeled to similar level as under the condition with active efflux seemed to suggest that although substrate were not extruded out of the cell, conversion between the three conformations was still possible. Since the proton relay pathway is intact, it is possible that translocation of protons could still occur, which drove the conformational rotation. Substrates could still migrate through the entire translocation pathway in AcrB and then be release back into the periplasm. The role of proton translocation in driving conformational rotation necessary for labeling was confirmed by the observation that the labeling of several sites was significantly weaker in AcrB_D407A_. These sites include S134C, N274C, D276C, R620C, and E673C. Since the D407A mutation has little effect on the overall structure of AcrB and does not disrupt its interaction with AcrA and TolC, it is reasonable to assume the observe decrease of labeling was a result of defect in proton translocation.

We observed the most dramatic changes of labeling of the most sites when AcrB was dissociated into monomers. While for some residues including R717, T676, L668, F664, D566, and F666, labeling in _CL_AcrB_Δloop_ was close to the level of labeling in _CL_AcrB, for the rest of the sites tested labeling was much less in _CL_AcrB_Δloop_. Sites labeled the least in _CL_AcrB_Δloop_ as compared to their levels of labeling in _CL_AcrB include Q89, N274, D276, and E673. An inspection of their locations in the structure of AcrB reveals that sites labeled to a level higher than 50% in _CL_AcrB_Δloop_ relative to their levels of labeling in _CL_AcrB unanimously located at the entrance and exposed sites of the external cleft, while sites labeled much weaker in _CL_AcrB_Δloop_ located in the binding pocket and the hidden part of the cleft (Figure 1B). The labeling results indicated that substrates could still access the lower cleft and external binding portal in monomeric AcrB, but could not enter into the deep binding pocket. Furthermore, we found that the interaction between AcrA and AcrB also depended on AcrB trimerization, as the level of AcrA cross-linked with AcrB_Δloop_ was much smaller than the amount cross-linked with wild type AcrB. The Interacting region for AcrA could be located close to the interface between two monomers of AcrB as it has been suggested in the assembly model proposed by Symmons and coworkers [38]. We cannot completely eliminate the possibility that local conformational change might have occurred in AcrB_Δloop_ and affected its interaction with AcrA, although the circular dichroism spectrum of AcrB_Δloop_ was very similar to that of the wild type AcrB [28].

Results from this study allows us to speculate why AcrB function as trimer. Trimerization is clearly required from the structural aspect for AcrB to dock properly with the trimeric outer membrane protein TolC. To investigate if each protomer in a trimer could function independently, Nikaido and coworkers had designed an elegant experiment to construct a covalently linked trimer [39]. The covalent trimer is fully functional, but loses most of its activity when the function of one protomer is disrupted. This result suggests that the functions of protomers are coupled in a trimer. Our result provided additional support for this picture. We found that while substrate could still bind to exposed sites at the entrance of the substrate translocation pathway, it failed to enter the deep binding pocket. Trimerization of AcrB is likely required to create three different and interlocked conformers in a functional unit to channel substrate unidirectionally out of the cell [40].

## Materials and Methods

### Creation of Knockout Out Strains

Strains MG1655 (F-, *λ* ^−^, *rph-1*), BW25113 (F-, *Δ (araD-araB)567, ΔlacZ4787*(::rrnB-3), *λ* ^−^, *rph-1, Δ (rhaD-rhaB)568, hsdR514*), and BW25113*ΔacrB* were obtained from the Coli Genetic Stock Center (Yale University). Single MG1655*ΔacrB* and combined MG1655*ΔacrABTolC* gene knockout strains were created using Quick & Easy *E. coli* gene deletion kit (Gene Bridges GmbH, Heidelberg, Germany) following the manufacturer’s protocol. In MG1655*ΔacrB*, *acrB* gene was replaced by a kan resistance cassette. In MG1655*ΔAcrABΔTolC*, the three genes were knocked out in two steps. Gene *tolC* was knocked out first followed by the removal of the kan resistance cassette introduced to replace the *tolC* gene. In the second step, *acrA* and *acrB* genes were deleted together following the same protocol. Colony PCR was applied to confirm that target genes have been knocked out.

### Protein Purification

Plasmids pQE70-AcrB, pQE70-AcrB_Δloop_, and the Cys-less version pQE70-_CL_AcrB, pQE70-_CL_AcrB_Δloop_ were constructed in a previous study [28]. They were transformed into *E. coli* BW25113*ΔacrB* for protein expression under the basal condition without induction. After grown overnight at 37°C with shaking, cells were collected with centrifugation and then disrupted using French press in a buffer containing 20 mM sodium phosphate (pH 7.5), 0.2 M NaCl, 10% glycerol, and 1 mM phenylmethanesulfonyl fluoride (PMSF). The membrane fraction was collected with ultra-centrifugation (100,000×g, 1 hour) and then solubilized in phosphate buffer containing 1% (w/v) n-dodecylb-D-maltoside (DDM). After centrifugation, protein in the supernatant was purified using Ni-nitrilotriacetic acid (NTA) superflow resin as described [28].

### Fluorescent Labeling

The two intrinsic Cys in AcrB were first replaced with Ala to create _CL_AcrB. _CL_AcrB has been shown by several studies to be fully functional [29], [41]. Using _CL_AcrB as the background, a single Cys was introduced to replace each residue as indicated one at a time. A thiol-specific fluorescent dye Bodipy-FL-maleimide was used in labeling. Bodipy-FL-maleimide labeling was conducted following the published method with slight modifications [26]. Briefly, BW25113*ΔacrB*, MG1655*ΔacrB*, or MG1655*ΔacrABtolC* containing indicated plasmids were cultured overnight at 37°C with shaking. Cells from 10 ml culture were collected through centrifugation at 4,000×g for 5 min, washed twice using 10 ml of phosphate buffer (50 mM potassium phosphate, 0.5 mM MgCl_2_, pH 7.0), and resuspended in 5 mL of the same buffer. Cell density was adjusted to OD_660_ of 3.5. Next, glucose and Bodipy-FL-maleimide were added to the cells to final concentrations of 0.4% and 6 µM, respectively. The mixture was shaken under room temperature for 40 minutes (200 rpm). Cells were then collected through centrifugation, washed with 5 ml phosphate buffer containing 0.4% glucose, and then washed again with 5 ml phosphate buffer.

Next, AcrB was purified as described with slight modifications [26]. Briefly, cells were lysed by sonication in ice water bath and centrifuged at 16,000×g for 20 minutes. The pellet was solubilized in a phosphate buffer (50 mM sodium phosphate, 0.2 M NaCl, 10 mM imidazole, pH 8.0) containing 2% DDM on ice for 2 hours. The sample was then centrifuged under 16,000×g for 20 minutes and the supernatant was collected. AcrB was incubated with Ni-NTA resins at 4°C for 2 hours with slow shaking. Resins were then washed with a buffer containing 50 mM sodium phosphate, 0.2 M NaCl, 40 mM imidazole, and 0.03% DDM (pH 8.0). Finally the protein was eluted with a buffer containing 50 mM sodium phosphate, 0.2 M NaCl, 500 mM imidazole, and 0.03% DDM (pH 8.0).

To test labeling of AcrB mutants with the over-expression of AcrA or TolC, plasmid pBAD33-AcrA or pBAD33-TolC were co-transformed with pQE70-AcrB_S134C_ or pQE70-AcrB_N274C_ into MG1655*ΔacrB*. The expression of AcrA or TolC was induced with the addition of 0.2% arabinose during the overnight incubation. Labeling was conducted as described above.

### Detection of the Labeling Signal

After protein purification, the eluted samples were resolved using SDS-PAGE on 8% gels. To detect the fluorescence labeling level by Bodipy-FL-maleimide, the gel was imaged using Typhoon Phosphorimager, with an excitation filter (488 nm) and an emission filter (510 nm). Then the gel was stained using Coomassie Blue R250 and imaged under white light. The intensities of the protein bands after staining revealed the protein concentration in the different lanes. The two images for each gel were analyzed and quantified using ImageJ [42]. The fluorescence intensity of each band was divided by its intensity in Coomassie blue stain to adjust for small variations in the quantity of sample loading. To determine the relative level of labeling, the concentration normalized fluorescence intensity for each site obtained in _CL_AcrB_Δloop_ was compared with the concentration normalized fluorescence intensity obtained in trimeric _CL_AcrB.

### Chemical Cross-linking

Chemical cross-linking was performed as described [36], [37]. Briefly, plasmid pQE70-AcrB, pQE70-AcrB_Δloop_, or the empty vector pQE70 was transformed into BW25113Δ*acrB* for protein expression under the basal condition. Cells were then harvested and treated with DSP. Extra DSP was quenched with a Tris buffer before cells were collected and lyzed for protein purification using Ni-NTA resin as described above. The eluted protein was incubated with dithiothreitol (DTT) before resolved using SDS-PAGE, followed by immunobloting with a polyclonal anti-AcrA or anti-AcrB antibodies as the primary antibodies, and an alkaline phosphatase-conjugated anti-rabbit antibody (Abcam, Cambridge, MA) as the secondary antibody. The protein-antibody conjugates were detected after staining using nitroblue tetrazolium chloride and 5-bromo-4-chloro-3′-indolyl phosphate p-toluidine.

### Drug Susceptibility Measurement

Activity of different AcrB constructs was determined by measuring the MIC of BW25113*ΔacrB* containing plasmids encoding each protein as described [28].
